# Case Study on the Use of Real-World Evidence in a Feasibility Assessment of a Randomized Controlled Trial Design to Support the Fulfillment of Pediatric Requirements with the FDA and EMA

**DOI:** 10.1007/s43441-026-00977-1

**Published:** 2026-06-19

**Authors:** Neal E. Storm, Christine Kubik, Ghislaine Priem, Min Kim, Tzu-Chieh Lin, Jeff Lange, Chelsea O’Connell, Anthony Glennane, Brian D. Bradbury, Mark J. Taisey

**Affiliations:** 1 Global Regulatory Affairs and Strategy, Amgen Inc., One Amgen Center Drive, Thousand Oaks, CA 91320 USA; 2 Center for Observational Research, Amgen Inc., One Amgen Center Drive, Thousand Oaks, CA 91320 USA; 3 Global Development, General Medicine, Amgen Inc., One Amgen Center Drive, Thousand Oaks, CA 91320 USA; 4 Department of Regulatory and Quality Sciences, School of Pharmacy, University of Southern California (USC), 1540 Alcazar Street, CHP 140, Los Angeles, CA 90089-9014 USA

**Keywords:** Prolia, Denosumab, Real-world evidence, Glucocorticoid-induced osteoporosis, Pediatric, Regulatory decision-making

## Abstract

**Introduction:**

Regulatory agencies may require industry sponsors of new drugs or biologics to conduct clinical trials that assess the benefits versus the risks of these new medicines in pediatric patients as a means of facilitating their development and availability for children when there is a medical need. Amgen Inc. (henceforth "the company") agreed to conduct a randomized, controlled trial (RCT) in pediatric subjects with Glucocorticoid-induced Osteoporosis (GiOP) under a Paediatric Investigation Plan (PIP) and Pediatric Study Plan (PSP) with the European Medicines Agency (EMA) and the United States (US) Food and Drug Administration (FDA), respectively, as one of the pediatric studies that were a condition of registration for Prolia (denosumab). Enrollment of pediatric subjects with GiOP into the agreed clinical study was exceedingly low despite the implementation of multiple mitigation measures. As a result, the company explored the use of real-world epidemiological analyses of the disease in children with GiOP for further insight into the clinical feasibility challenges with the RCT design.

**Materials and Methods:**

We initiated a phase 3 randomized, double-blind, placebo-controlled, parallel-group study to evaluate the safety and efficacy of denosumab in pediatric subjects with GiOP (Study 20140444). Following difficulties in recruiting we re-assessed the enrollment potential and overall clinical trial feasibility of Study 20140444 using real-world epidemiological analyses evaluating the size of the pediatric GiOP population using real-world data (RWD) from 3 different databases: (1) the US MarketScan, (2) the United Kingdom (UK) Clinical Practice Research Datalink (CPRD), and (3) the IQVIA Disease Analyzer (Germany).

**Results:**

The results from the RWD show that very few pediatric patients met the clinical diagnosis of GiOP, irrespective of the database used. In sum, approximately 1 eligible subject per 1,000,000 people per year was estimated to be available for Study 20140444.

**Conclusions and Recommendations:**

The results suggest a lack of feasibility of conducting an adequate and well-controlled randomized trial in a population of pediatric patients with GiOP. The results also called into question the feasibility of other types of clinical trial designs, including potential single-arm or hybrid study designs, to fulfill the regulatory requirements. Recommendations include considering the use of RWD-based epidemiological analyses prior to agreeing with health authorities to conduct postmarketing required pediatric studies or studies in other rare diseases. This approach to clinical trial feasibility using RWD helps ensure research goals are balanced with the needs of the individual patient, further ensuring the ethical conduct of clinical trials.

**Clinical Trial Registration (Study 20140444):**

ClinicalTrials.gov / NCT03164928 / URL: https://clinicaltrials.gov/search? term=NCT03164928. EudraCT / 2016-003083-39 / URL: https://www.clinicaltrialsregister.eu/ctr-search/search? query=2016-003083-39.

## Introduction

Regulatory agencies may require industry sponsors of new drugs or biologics to conduct clinical trials that assess the benefits versus the risks of these new medicines in pediatric patients as a means of facilitating their development and availability in children when there is a medical need. Pediatric study requirements exist because children are not simply small adults; they possess distinct physiological, developmental, and pharmacological characteristics that affect how their bodies metabolize medications [1]. Historically, drugs were rarely tested in children, leading to widespread “off-label” prescribing based on adult data, which increased the risk of unexpected adverse drug reactions, toxicity, or ineffective treatment [2]. Thus, the pediatric regulatory requirements of the FDA and EMA exist to ensure pediatric patients have the potential of receiving access to the same evidence-based treatments as adults.

In the US, the Pediatric Research Equity Act (PREA) requires any new application or supplement to a marketing authorization application to provide a pediatric assessment in accordance with an agreed upon Pediatric Study Plan (PSP) or to seek a waiver or deferral for such pediatric studies. The PSP provides an outline of the pediatric study or studies that the drug developer plans to conduct to confirm the suitability of drug usage to cover a specific pediatric clinical need [3]. In the EU, the Paediatric Regulation (Regulation (EC) No. 1902/2006, as amended) requires industry sponsors to have an agreed Paediatric Investigation Plan (PIP) in place, comprising studies that companies plan to conduct involving children to support the authorization of medicines for children, unless exempted because of a deferral or waiver. Like the objectives of the PREA, the Paediatric Regulation is intended to improve and better protect the health of European children by facilitating the development and availability of medicines for children aged 0 to 17 years [4]. 

Prolia^®^ (denosumab, 60 mg/mL every 6 months [Q6M]) is a fully human monoclonal antibody that binds and neutralizes the RANK ligand (RANKL). Inhibiting RANKL in patients with osteoporosis results in reduced osteoclast numbers and function, thereby decreasing bone loss and increasing bone mass and strength in both cortical and trabecular bone [5]. Initial approval for the treatment of women with postmenopausal osteoporosis (PMO) who are at increased/high risk of fracture was based on 3-year data from a randomized, double-blind, placebo-controlled, phase 3 multiregional clinical trial (MRCT) [5]. Clinical trial evidence demonstrates that treatment with Prolia significantly reduces the risk of vertebral, nonvertebral, and hip fractures and, in an open-label extension study, continues to increase bone mineral density (BMD) over 10 years at key skeletal sites without evidence of a therapeutic plateau [6]. In addition, the incidence of fractures at spine, hip, and non-spine sites remains low with continued treatment for up to 10 years [6].

Following the European Medicines Agency (EMA) Committee for Medicinal Products for Human Use (CHMP) issuance of a positive Opinion on May 26, 2010 for the treatment of PMO, followed shortly thereafter by a US Food & Drug Administration (FDA) approval on June 1, 2010, supplemental indications were added to the labeling to treat bone loss in men with osteoporosis who are at increased/high risk for fracture [7] and bone loss in patients undergoing hormone ablation therapy (HALT) for prostate or breast cancer [8]. In 2018, Prolia was registered for use in adults to treat glucocorticoid-induced osteoporosis (GiOP) in men and women at increased/high risk for fracture in the EU and the US [9, 10]. Following the establishment of benefit: risk in patients with adult GiOP, the company proceeded with evaluating the benefits versus the risks of Prolia in pediatric GiOP [11]. Clinical studies supporting these supplemental indications, including GiOP and pediatric GiOP, were designed in accordance with FDA recommendations that first require the demonstration of fracture efficacy in a RCT (generally, the clinical trial supporting the registration of the initial indication of PMO) to validate the use of the BMD endpoint as the primary endpoint for other indications [12]. The position of the EMA was similar to that of the FDA in that after therapy is approved for the treatment of osteoporosis in PMO, a BMD bridging study in the secondary osteoporosis setting using the same dosing regimen may be sufficient for approval provided that the duration of the study is at least 1 year, the dose is justified, the eligibility criteria generate a similar fracture risk as in women with PMO, and the magnitude of changes in BMD versus placebo is similar to that in women with PMO [13]. Decreased BMD has been described in various pediatric disorders that require glucocorticoid (GC) treatment, such as asthma, juvenile rheumatoid arthritis, inflammatory bowel disease, systemic lupus erythematosus, and organ transplantation [14]. At that time, GiOP was considered the second most common form of osteoporosis and the most common iatrogenic form of the disease [15], and the prevalence of oral GC use was estimated to be between 0.2% and 1% in the general population. With GiOP, rapid bone loss (6–12% of BMD) occurs during the first 6–12 months of GC treatment, and the risk of fracture increases to 75% within the first 3 months of therapy [16]. Thus, a medical need appeared to exist for a treatment such as Prolia that could be provided once every 6 months for both adults and children.

In addition to developing Prolia for the treatment of adult disease, the company received, upon approval of the GiOP indication, a postmarketing requirement (PMR #3422-1) study to evaluate the benefits versus and the risks of Prolia in pediatric GiOP in a phase 3 randomized, double-blind, placebo-controlled, parallel-group study to evaluate the safety and efficacy of denosumab in pediatric GiOP (Study 20140444; NCT03164928). Although the mechanism of action of anti-resorptive medicines is similar in adults and in children, there are several differences between the two in terms of the pathogenesis of bone disease and the effects of treatment on the growing bone. Also, the dosing and exposure-treatment effect ratio in children may be different thus necessitating the generation of PK/PD and safety data in children. These factors may not allow full extrapolation and, therefore, clinical studies in children are normally necessary to support a proper evaluation of benefit:risk of a particular therapy in children [17].

Approximately 150 subjects were to be enrolled. This enrollment target was deemed necessary to achieve adequate power to detect differences in BMD between the treatment arms. The subjects were randomized to receive either 1 mg per kg body weight denosumab (up to a maximum of 60 mg) subcutaneous (SC) Q6M or matching placebo SC Q6M. Like the clinical BMD bridging studies conducted to support the adult line extension indications, the primary objective of the study was to evaluate the effect of denosumab on the lumbar spine BMD Z-score as assessed by dual X-ray absorptiometry dual X-ray absorptiometry (DEXA) at 12 months in children 5 to 17 years of age with GiOP. It was determined with regulators that Prolia would pose safety concerns in pediatric patients under 5 years of age due to the high rates of skeletal growth and the potential for Prolia to adversely affect long-bone growth and dentition in this age group [18]. Hence, a waiver for this pediatric study requirement for ages 0 to 4 years was granted, and for the age group of 5–17 years, the pediatric study was deferred [18]. Importantly, no formal regulatory guidance or guidelines existed for the development of therapeutics for either GiOP or pediatric GiOP at the time of study design, the company considered the Recommendations for Registration of Agents for the Prevention and Treatment of GiOP provided in the 2008 Consensus Statement from the Group for the Respect of Ethics and Excellence in Science (GREES) [19].

An EMA PIP was initially agreed upon with the Paediatric Committee of the European Medicines Agency (PDCO-EMA) in April 2013 (EMA-000145-PIP02-12) [20] and agreement with the US FDA on an initial PSP occurred in June 2017 [21]. With the FDA approval of the adult GiOP supplement in May of 2018, FDA issued a postmarketing requirement for Study 20140444, as a condition of continued licensure in the US. The requirements for the study design were negotiated to be consistent between the PSP and PIP; both required the study’s last patient last visit (LPLV) to be completed by December 2022. Per ClinicalTrials.gov and EudraCT, the clinical trial was completed on December 20, 2023 (NCT03164928).

### A Question of Feasibility

The pediatric clinical trial for GiOP was initiated in June 2017 once the benefit:risk profile had been established in the adult GiOP population. The first subject was enrolled in May 2018 and the enrollment of pediatric subjects with GiOP was observed to be slow early in the study process. Persistent delays in study enrollment resulted in only 16 subjects of a target enrollment of 150 being enrolled at 11 of the 36 clinical sites that were activated globally (at the time feasibility concerns were raised to health authorities). Understanding the reasons for the delays and expeditiously informing the FDA and EMA of the enrollment challenges were both critically important.

A clinical feasibility analysis of the study sites revealed difficulties in identifying appropriate per-protocol subjects, resulting in a very small number of available subjects that could be enrolled resulting in a high screening failure rate. Consistent with treatment guidelines, the target population of the study was required to include a subject population that met the clinical diagnostic criteria of GiOP [22, 23] with a specific inclusion criterion for evidence of at least 1 vertebral compression fracture of Genant grade ≥ 1, OR presence of both a clinically significant fracture history and a lumbar spine BMD Z-score ≤ − 2.0. The majority of the subjects screened for this study (~70–75%) did not meet these specific inclusion criteria and therefore could not be enrolled in the study.

The company undertook several due diligence measures to mitigate the issue of low enrollment. Given that both the adult and pediatric GiOP population is diverse and stems from various disease areas, the company sought to actively recruit investigators at pediatric endocrinology, pediatric rheumatology and pediatric neurology clinics. Because enrollment was extremely limited at the original clinical trial sites, additional clinical sites/countries were added globally to expand the global footprint of the trial to access patients who met the protocol criteria. Initially, 30 sites were activated in 12 countries. However, because enrollment was extremely limited at the original sites, 9 additional countries were added (leading to an increase in the number of sites from 30 to 48). The company also attempted to increase subject enrollment by implementing referral networks and study advertisements. Despite a multitude of efforts, subject enrollment did not meaningfully improve. As a result of the difficulties in identifying patients per protocol, several clinical sites globally declined participation in the study, with many active sites requesting that the company terminate their participation because of the limited number of available subjects. The empirical evidence pointed to the availability of very few subjects globally who met the clinical definition of GiOP per the ISCD guidelines and the inclusion criteria required by the FDA and EMA, but definitive evidence was needed.

Several hypotheses relating to the study design were offered by the clinical investigators themselves to explain the smaller than anticipated patient population. First, the study design itself may have been a barrier to enrollment since it relied on the use of a placebo rather than active control, similar to that of the adult trial in GiOP. In addition, the inclusion criteria requiring prior history of osteoporotic fracture was deemed a potential barrier to enrollment, though it was ultimately agreed this could not be modified because treatment with antiresorptives is not recommended in pediatric patients with GiOP unless they have a prior history of fracture [18]. Finally, it was hypothesized that the per-protocol population was decreasing because of an increase in the use of biologics rather than GC use in children to minimize the potential for adverse effects. While the American College of Rheumatology (ACR) has developed clinical guidelines for the treatment of GiOP [24], there are no generally accepted guidelines that are used consistently in pediatric GiOP patients. The ACR guidelines acknowledge that limited clinical data exist related to the treatment of pediatric GiOP patients [25]. One plausible interpretation of this is that the paucity of patients who meet the pediatric GiOP clinical definition has made clinical research difficult to conduct. This hypothesis is potentially corroborated by the observation that pediatric GiOP is rarely reported as an off-label indication in the postmarketing setting, as reported through the company's routine pharmacovigilance.

The company required additional tools and data to understand the true size of the contemporaneous pediatric GiOP population. Once the specific issues that prevented the clinical trial from being conducted were diagnosed, credible hypotheses supported by robust evidence could be presented to regulatory authorities to support regulatory decision-making.

## Materials and Methods

To assess the size of the pediatric GiOP population, an evaluation of trends in pediatric GC use over time using real-world data (RWD) was performed using 3 different databases: (1) the US MarketScan, (2) the UK Clinical Practice Research Datalink (CPRD), and (3) the IQVIA Disease Analyzer in Germany. These 3 databases were selected to comprise a representative population of patients from the countries regulated by the FDA and the EMA.

The prevalence of pediatric GiOP was estimated by selecting pediatrics (≥ 1 and < 18 years of age) diagnosed with conditions commonly treated with systemic GC. Pediatrics with a prevalent diagnosis of rheumatoid arthritis, inflammatory bowel disease (Crohn’s disease and ulcerative colitis), psoriasis, immune thrombocytopenic purpura, multiple sclerosis, nephrotic syndrome, systemic lupus erythematosus, muscular dystrophy, neuropathy and asthma were evaluated for long-term use of steroids and evidence of osteoporotic/fragility fracture annually from 2013 to 2018. Long-term steroid use was defined (using pharmacy claims data) as 90 or 180 continual days (allowing for gaps of 7 days between prescriptions) of GC use over a 1-year period. Analyses were specified for 90 or 180 days to be consistent with the guidance in the 2010 ACR Recommendations for the Prevention and Treatment of GiOP that a patient would be treated with GC for at least 3 to 6 months before developing a fracture risk associated with GC use [24]. Annual estimates of the average number of continual and total days of GC use (over a 1-year period) were obtained for children diagnosed with the 10 conditions (listed above) who in the year prior, experience a fragility fracture.

In the US, we used the MarketScan database, which includes children who are covered under their guardians’ commercial health plan rather than those covered under publicly funded programs (e.g., Medicaid/Children’s Health Insurance Program). The MarketScan database provides data on inpatient and outpatient medical and outpatient prescription drug coverage for ∼196 million patients and their dependents (∼25 million annually) who receive health care coverage from employer-sponsored plans in the US.

In the UK, we used the Clinical Practice Research Database (CPRD), which includes children in the primary care setting. The CPRD database provides longitudinal data from the electronic medical records (EMRs) of ∼5 million active patients (∼14 million cumulative patients) who obtained care in a primary care/general practitioner (GP) setting in the UK.

 In Germany, we used the IQVIA Disease Analyzer, an electronic healthcare records (EHR) database which also includes children in the primary care setting. All three systems are population-based data systems that capture all age groups, including children. The IQVIA Disease Analyzer database provides longitudinal data extracted from the EMRs of ∼14 million active patients (in the past 3 years) who obtained care in a primary care/general practitioner setting.

Using RWD from 3 different countries aids in the evaluation of trends in pediatric GC use in populations relevant to European and U.S. regulators. Age and sex distribution in these databases, which comprise 7.4% of the US population and approximately 5% of the UK and German populations, are similar with the general population. Differences in the inclusiveness of the healthcare systems emerge between the children covered in the EU countries versus the US. The MarketScan database only includes children that are covered under their guardians’ commercial health plan, missing out on those covered under publicly funded programs (e.g., Medicaid/Children’s Health Insurance Program). In the UK and Germany, the vast majority (close to 90%) of the population receives health insurance through publicly funded programs. the CPRD database only captures children in the primary care setting, the age distribution of the database closely follows the age distribution of the entire UK population, providing further evidence of the generalizability of the CPRD analysis. Similarly, the Germany EMR database only captures children in the primary care setting. However, the age distribution of the German national population is much younger compared to the IQVIA Disease Analyzer database.

In this assessment of the enrollment potential and overall trial feasibility of Study 20140444, several key methodologic steps were considered essential for a robust evaluation of this RCT (Table 1). Consistent with recommendations described in recent literature, we systematically integrated insights and evidence derived from RWD to facilitate a deeper understanding of the specific feasibility challenges associated with Study 20140444 [26, 27].

**Table 1:** Methods and considerations used for evaluating randomized-controlled trial feasibility

Method	Considerations
i) Define clinical phenotype	•Address any meaningful differences in the definition of disease that likely impact the size of the eligible population; screen failure rate (as defined by clinical guidelines or regulatory authority guidance) prior to conducting analyses in RW databases
ii) Characterize epidemiology of indication and estimate size of target population	•Conducted by country/region to inform clinical site selection
iii) Characterize patterns of clinical care and benchmark the standard of care (SoC)	• Map the current treatment landscape and anticipate potential future changes in SoC (e.g., hypothesized transition from glucocorticoids to biologics). • Ensure comparator arms are aligned with prevailing SoC. *°Note: A placebo arm may not be appropriate in pediatrics due to potential ethical or parental acceptability concerns.*
iv) Evaluate key study design elements/enrollment criteria (inclusion/exclusion criteria)	•Significance for informing: ° Size of the eligible population ° Anticipated screening failure rate
v) Additional considerations from the literature	• Estimate the size of the target population by country to support the selection of sites • Devise strategies to enhance equitable access and representation • Estimate expected clinical event rates to support power calculations • Esimate range of expected event rates for safety assessments • Inform site selection and recruitment rates using prior studies

By implementing analyses of RWD into our overall approach, we sought to provide a comprehensive package that could inform a regulatory decision regarding the feasibility of conducting a pediatric GiOP clinical trial.

## Results

In the US MarketScan analyses, the number of children diagnosed with any of the 10 qualifying indications decreased from more than 400,000 in 2013 to approximately 290,000 in 2018. Within this group, GC use decreased from 33% in 2013 (*n* = 132,143) to 22% in 2018 (*n* = 64,543). Osteoporotic fractures within this population were rare, ranging from 1.0% (*n* = 3662) to 1.7% over the time frame. Among the subjects with osteoporotic fractures, the number of children who used GCs continuously for at least 90 days was low, ranging from 24 (0.35%) to 102 (1.82%) in any given year. The annual counts decreased even further (range: 13 to 42 children) if the GiOP definition was restricted to 180 days of continual GC use. Over a 1-year period, the median number of days of GC use ranged from 18.0 to 30.0 among children with any of the 10 disease indications who had recently experienced osteoporotic fracture. Selecting for each child’s longest stretch of continual GC use, the median number of days of continuous GC use over a 1-year period ranged from 13.0 to 19.0 within this population.

Similar results were observed in the UK CPRD database. The number of children with prevalent disease (any of the 10 indications) decreased substantially from 2013 (*n* = 90,569) to 2018 (*n* = 39,479). Among children with prevalent disease, approximately 20% had received any GC in any given year, and a much smaller proportion (range: 0.39% to 0.49%) had recently experienced an osteoporotic fracture from 2013 to 2018. Among those who had experienced an osteoporotic fracture, only 0 to 6 children (in any given year) had used GCs for ≥ 90 continuous days, and 0 to 5 children had used GCs continuously for ≥ 180 days from 2013 to 2018. Over a 1-year period, the median number of days of GC use ranged from 8 to 15 days for children who had experienced an osteoporotic fracture from 2013 to 2018. Selecting for each child’s longest stretch of continual GC use, the median number of days of continuous GC use over a 1-year period ranged from 5 to 15 days within this group.

According to the IQVIA Disease Analyzer (Germany), the number of children with prevalent disease ranged from 20,538 in 2013 to 10,932 in 2018. Within this group, 12% to 14% had received any GC, and 0.02% to 0.05% (range: 6 to 11 children) had experienced recent osteoporotic fracture. Among children with any osteoporotic fracture, the number with 2 GC prescriptions separated by ≤ 90 days ranged from 0 to 3 in any given year (from 2013 to 2018). Within this population, the median number of GC prescriptions over a 1-year period was 1.0.

Table 2 presents the sample size estimates from the US MarketScan, UK CPRD and IQVIA Disease Analyzer (Germany) EMR systems as natural frequencies to provide an absolute number of the population for easy comparison across the three data sources. In sum, approximately 1 eligible subject per 1,000,000 people per year was estimated to be available for Study 20140444.

**Table 2:** Presentation of sample size estimates in 2018 from US MarketScan, UK CPRD and IQVIA Disease Analyzer (DE)

	US MarketScan	UK CPRD	IQVIA Disease Analyzer (DE)
Annual population	25,000,000	5,000,000	2,000,000
Pediatrics with inflammatory Dx per year	290,582	39,479	10,932
With any glucocorticoid use*	64,543 (22.2%)	8115 (20.6%)	1446 (13.2%)
With any fracture*	3662 (1.3%)	176 (0.4%)	27 (0.2%)
and with glucocorticoid use ≥ 90 days**	60 (1.7%)	2 (1.1%)	N/A
and with glucocorticoid use ≥ 180 days**	13 (0.4%)	2 (1.1%)	N/A
and with 2 prescriptions for glucocorticoids ≤ 90 days**	N/A	N/A	1 (3.7%)
Potentially eligible subjects per 1 million population	0.5	0.4	0.5

*Denominator includes the number of pediatric patients with inflammatory conditions

**Denominator includes the number of pediatric patients with inflammatory conditions and with any osteoporotic fracture in the past year

Consistent with the results of the traditional clinical feasibility analyses, the results from the RWD show that very few subjects met the clinical diagnosis of GiOP. These results precluded not only the possibility of conducting an adequate and well-controlled RCT but also other types of clinical trial designs, including potential single-arm or hybrid approaches, to address any important gaps in pediatric evidence that may remain. These results appeared consistent with the observations of the study investigators and available regulatory precedents that the company had researched from the US and the EU, including Aclasta (zoledronic acid) (NCT00799266) and pamidronate (NCT00022841) [28, 29]. Importantly, the company was able to demonstrate conceptual replication of the findings across the 3 data systems/countries where the same question was addressed with different data in geographies relevant to both the US FDA and EMA [30]. In addition, the EMA’s Data Analytics and Methods Taskforce conducted an independent analysis of RWD (using the IMS France and IMS Germany data sources) to evaluate whether pediatric patients exposed to long-term prescription of glucocorticoid-containing products exist in sufficient numbers to support a pediatric trial in GiOP. The EMA results were similar to those obtained by the company, suggesting the number of pediatric patients exposed to long-term glucocorticoids is low and decreasing [31]. A relatively small number (< 60 in 10,000) of pediatric patients have a long-term GC exposure and the incidence of osteoporosis and/or fracture in those with long-term GC exposure is frequently under 1 per 100 person-years, corresponding to fewer than five incident cases per year, per database [31]. The results of the EMA Data Analytics and Methods Taskforce independently suggest that recruitment for a clinical trial in pediatric GiOP is challenging [31].

Since Study 20140444 was no longer able to test the central scientific hypothesis under the protocol, the company sought to use these data to seek release from its pediatric commitments to conduct a pediatric GiOP clinical study on the grounds that there were too few children with the disease or condition, which is a key ground for supporting such a request in the Pediatric Research Equity Act [PREA] Section 505B(a)(4)(A)(i)).

### Potential Limitations of the Use of RWD

The evidence package submitted to regulators provided population-based assessments of the prevalence of GiOP in pediatric patients across multiple countries. The strength of this evidence should be considered in light of potential limitations of the data, including changes in coding practices over time that may contribute to varying prevalence estimates. Also, the degree to which the populations captured in the databases are fully representative of the GiOP population may have been impacted by the fact that the databases did not include those covered by Medicaid and the Children’s Health Insurance Program (CHIP) in the US or the non-primary care population in the EU. However, these limitations do not influence our interpretation of the results given (1) the trends were consistent across the 3 databases, (2) the prevalence of GiOP in the pediatric setting was observed to be rare and (3) evolving treatment patterns may have further reduced the availability of patients who met the study criteria.

### Regulatory Outcomes with the EMA and FDA

Following the submission of the RWD-based feasibility evaluation, the company obtained agreement from the EMA and the FDA in December 2020 for early termination of the pediatric GiOP study as a result of a significant decrease in the prevalence of pediatric GiOP. In accordance with the agreements with FDA and EMA, Study 20140444 discontinued enrollment but allowed the 24 enrolled subjects to complete the 3-year study. This allowed valuable information to be obtained in this rare pediatric population of GiOP patients treated with 1 mg/kg denosumab on an every 6-month dosing regimen. The study was completed in December 2023 and the results were subsequently submitted to health authorities. The EU PIP was confirmed to be compliant on December 13, 2024 [32], and the US FDA postmarketing commitment for the pediatric study was deemed fulfilled on May 18, 2025. After a US supplemental biologics license application approval, language was added to Section 8.4 (Pediatric Use) of Prolia’s US prescribing information (USPI v.28) summarizing the study design and available results from Study 20140444. Prolia remains unapproved for use in pediatric patients:

*“Safety and effectiveness were not demonstrated for the treatment of glucocorticoid-induced osteoporosis in one multicenter*,* randomized*,* double-blind*,* placebo-controlled*,* parallel-group study conducted in 24 pediatric patients with glucocorticoid-induced osteoporosis*,* aged 5 to 17 years*,* evaluating change from baseline in lumbar spine BMD Z-score.”* [33].

Similarly, the following statement was added to Section 5.1 (Pharmacodynamic properties, subsection on *Paediatric population*) of the Prolia EU Summary of Product Characteristics (SmPC):

*“In one multicentre*,* randomised*,* double-blind*,* placebo-controlled*,* parallel-group study conducted in 24 paediatric patients with glucocorticoid-induced osteoporosis*,* aged 5 to 17 years*,* evaluating change from baseline in lumbar spine BMD Z-score*,* safety and effectiveness were not established hence Prolia should not be used for this indication.”* [34].

In addition to the regulatory outcomes described above for Prolia, the same RWE accepted by the FDA, and reconfirmed by the EMA’s Data Analytics and Methods Taskforce, contributed to the removal of a similar pediatric GiOP PIP requirement for another osteoporosis product, Evenity (romosozumab). For Evenity, a PIP was agreed in March 2016 (EMA decision P/0066/2016) to conduct 2 studies with pediatric GiOP patients [35]:

- *“Study 4 - A Randomised*,* double-blind*,* placebo-controlled*,* ascending multiple dose study to evaluate the safety*,* pharmacokinetics and pharmacodynamics or romosozumab in paediatric patients from 10 to less than 18 of age with glucocorticoid inducted osteoporosis.”*
- *“Study 5 – Randomised*,* double-blind*,* placebo-controlled study to evaluate the efficacy and safety of romosozumab compared to placebo in paediatric patients from 10 to less than 18 years of age with glucocorticoid-induced osteoporosis.”*

Using the same RWE developed for Prolia, the company's business partner in the EU interacted with the EMA in 2021 with the goal of releasing their pediatric study requirements for Evenity. As with Prolia, EMA agreed the real-world epidemiology showed clear evidence that pediatric patients with GiOP are, in fact, very rare, which makes recruitment in a pediatric GiOP clinical study infeasible. EMA acknowledged that contemporary EU clinical guidelines have led to a reduction in the long-term prescription of glucocorticoids in the EU over the past decade, and their use in this population is very low. On the basis of these considerations, the EMA PDCO agreed to remove Studies 4 and 5 from the Evenity EU PIP on December 31, 2021 (P/0551/2021) [36].

## Discussion and Conclusion

As described in Hernandez RK, Critchlow CW, Dreyer, N, et al. [37] and Storm, Chang, Lin et al. [38], the range of potential uses for RWD/RWE in regulatory decision-making is still evolving and expanding. In this case study, RWD was used to add further support to the results of traditional assessments of clinical trial feasibility to justify early discontinuation of a pediatric GiOP study and to fulfill pediatric requirements with the FDA and the EMA on the grounds that there were too few children with the disease or condition to study, a key element needed to demonstrate study infeasibility under the Pediatric Research Equity Act [PREA] Section 505B(a)(4)(A)(i)). This approach of augmenting traditional clinical trial feasibility with RWD is consistent with the recommendations developed by the Clinical Trials Transformation Initiative (CTTI) for using RWD when planning eligibility criteria and recruiting clinical trials. Recently, Gatto and Campbell [26] reported instances where the use of RWE may have averted challenges to trial feasibility, such as where a sponsor relied on the pivotal trial design used to support the approval of another treatment for a similar indication but did not monitor changes in standard of care, which had in fact changed appreciably. Regulatory guidance on using RWD to assess RCT feasibility is currently limited and the China Center for Drug Evaluation at the National Medical Products Administration (NMPA-CDE) provides what is perhaps the most specific language endorsing RWD for feasibility to guide clinical study design in its Guidelines for the Use of Real-World Evidence to Support Drug Research & Development and Evaluation [39]. The CDE’s guidance explicitly states that RWE can “provide a valid reference for inclusion and exclusion criteria, parameters for sample size estimation, and the determination of noninferiority margins”, suggesting that RWE can be “helpful for judging the reasonability of designs.”

This case study contributes to the literature supporting the use of RWD strategies in situations where there may be uncertainty regarding the size of the population to be studied and where clinical equipoise may be in question. While this analysis evaluated how RWD can be used to augment an assessment of clinical trial feasibility in a specific disease setting such as pediatric GiOP, the findings have broader policy and public health implications. The methods can be used to rule out study designs for specific rare orphan disease or populations, where conducting RCTs have historically been associated with difficulties. Thus, the use of RWD feasibility strategies may facilitate the proper evaluation of medicines in children and ultimately help to ensure appropriate use in clinical settings.

The value of industry drug sponsors providing epidemiological data to supplement available evidence to facilitate appropriate regulatory decision-making cannot be overstated. Regulatory authorities should require RWD to be used during clinical feasibility estimates of the size of the per-protocol patient population prior to conducting clinical studies, and similarly, good practice may involve health authorities carrying out analyses confirming the results of industry sponsors, as EMA had done in this case study. Conceptual replication by the health authority may reduce common concerns regarding the reliability and quality of RWD in regulatory decision-making by confirming that findings are robust across different study conditions, populations, or data sources and strengthens the confidence in the findings. In this regard, this approach may add another potentially useful application for the DARWIN EU (Data Analysis and Real World Interrogation Network) tool that the EMA is developing to provide high-quality real-world evidence on the use, safety and effectiveness of medicines across the EU. This approach may be particularly helpful for studies conducted in support of regulatory commitments and in rare and sensitive patient populations when limited natural history data exist for the disease, and particularly in situations where the treatment landscape may be rapidly evolving. Another recommendation is for sponsors to consider conducting such epidemiological analyses before interacting with health authorities on clinical development plans, potentially increasing the efficiency of RCT designs and/or minimizing the burden of regulatory commitments. Importantly, such measures may reduce the need to initiate studies and expose patients to investigational treatments when the evidence suggests that the standard of care is evolving. This approach of using RWD to evaluate clinical trial feasibility may help ensure that research goals are balanced with the needs of the individual patient, further ensuring the ethical conduct of clinical trials. 

## Data Availability

Qualified researchers may request data from Amgen clinical studies. Complete details are available https://wwwext.amgen.com/science/clinical-trials/clinical-data-transparency-practices/clinical-trial-data-sharing-request/.
